# Use of Cytopoint in the Allergic Dog

**DOI:** 10.3389/fvets.2022.909776

**Published:** 2022-07-19

**Authors:** Margaret Gober, Andrew Hillier, Manuel A. Vasquez-Hidalgo, Deborah Amodie, Martha A. Mellencamp

**Affiliations:** ^1^Zoetis, Inc., Parsippany, NJ, United States; ^2^Department of Animal Sciences, North Dakota State University, Fargo, ND, United States

**Keywords:** lokivetmab, allergic dermatitis, dogs, pruritus, anti-IL-31

## Abstract

Allergic dermatitis is the most common type of skin disease in dogs. Of all dogs, 20 to 30% present with some type of allergic dermatitis. Pruritus is one of the most important signs of allergic dermatitis and is often the most challenging to control. Interleukin-31 (IL-31) has been found to be one of the main initiators of pruritus in dogs with allergic dermatitis. Cytopoint®, a caninized monoclonal anti-IL-31 antibody, has been shown to be effective for the treatment of dogs against allergic dermatitis and atopic dermatitis. US label indication. A recent retrospective study reported that Cytopoint achieved treatment success in 87.8% of the cases with allergic dermatitis. No prospective cohort studies have been performed investigating the effects of Cytopoint in dogs with allergic dermatitis using the dosing protocol prescribed on the product label in the United States. In this study, our objectives were to assess the efficacy of Cytopoint for treatment of canine allergic dermatitis of variable etiologies and management of the associated pruritus, and add to the body of evidence available to the veterinarian as they make treatment recommendations. Dogs included in this study had moderate to severe pruritus according to the Pruritus Visual Analog Scale (PVAS; ≥ 50 mm) and a history of likely continuation of pruritus at the time of presentation. On day 0, investigators recorded the initial body weight and every patient received one dose of Cytopoint (minimum 2 mg/kg SQ) and an isoxazoline product for parasite control. Treatment success for this study was defined as a ≥20 mm reduction in PVAS from Day 0. On Day 7, 94% of the dogs had achieved treatment success. On Day 28, 98% had achieved treatment success and cumulatively by day 56, 100% of the dogs achieved treatment success. This prospective study provides evidence that Cytopoint effectively treats dogs with allergic dermatitis of different types and the associated pruritus.

## Introduction

Allergic dermatitis, including atopic dermatitis (CAD), contact allergy, flea allergy dermatitis, and cutaneous adverse food reactions, is the most common type of skin disease in dogs (1–3). Of all dogs, 20 to 30% present with some sort of allergic dermatitis with 10 to 15% showing atopic dermatitis (1, 2, 4, 5). One of the hallmarks of allergic dermatitis is pruritus.

The pathogenesis of allergic dermatitis is complex and involves both the animal's genetic background, and exposure to environmental allergens (6). In atopic dermatitis, dogs are predisposed to recurrent skin infections, have a dysregulated immune response and have dysfunction of their skin barrier which allows allergens to enter the body through the skin and initiate abnormal immunological reactions (6). These reactions involve a myriad of different cytokines (6) including interleukin-31 (IL-31) which has been found to be one of the main inducers of pruritus in dogs with atopic dermatitis (7). Interleukin-31 binds to receptors on peripheral neurons likely activating pruritogenic signals in peripheral nerves (8, 9). More recently the role of IL-31 in the immune functions and on keratinocytes has started to emerge (10).

Systemic treatments to control pruritus in allergic and atopic dogs have usually included glucocorticoids and/or ciclosporin (11, 12). For long-term therapy, ciclosporin can have a slow onset of activity and an increased cost to the pet owner. Glucocorticoids, while effective in the short-term control of allergic pruritus and inflammation, can have secondary adverse reactions with significant morbidity, making them a less than ideal choice for long-term treatment (11). Targeted treatments for IL-31 include oclacitinib which selectively inhibits JAK 1 dependent cytokines (Including IL-31, 2, 4, 6, 8, 13) (10) and Cytopoint®, a caninized monoclonal anti-IL-31 mAb (13). The proven efficacy, safety profile, increased compliance and no contraindications make Cytopoint an attractive choice for the treatment of allergic and atopic dermatitis and the pruritus that is often present.

In 2018 in the United States, Cytopoint® was granted an extended label indication for its use in dogs with allergic dermatitis, in addition to the original claim for use in dogs with atopic dermatitis. The dosing protocol for both disease conditions is a minimum of 2 mg/kg administered subcutaneously every 4-8 weeks (14). A retrospective study published in 2018 reported that lokivetmab achieved treatment success in 87.8% of the cases with allergic dermatitis (15). The same study reported that the type of allergic dermatitis was not correlated with the effectiveness of lokivetmab (15). A prospective study (16) using the European labeled dose of Cytopoint(1-3.3 mg/kg) for treatment of pruritus associated with allergic dermatitis was recently completed. Results of this study showed significant reduction of owner-assessed pruritus after treatment with lokivetmab compared to placebo (saline) control. However, to our knowledge, no prospective cohort studies have been performed investigating the effects of Cytopoint in dogs with allergic dermatitis at the US label dose. In this study, our objective was to assess the efficacy of Cytopoint for management of pruritus in dogs with allergic dermatitis of variable etiology.

## Materials and Methods

This was an open prospective study of pet dogs administered Cytopoint at US label dosing (minimum 2 mg/kg) with a single injection at Day 0.

### Patient Selection

Investigators from eight general practices in the Northeast, Southeast and Midwest United States participated in this study. The practices were a mix of private (6) and corporately owned (2) general companion animal practices. Data was collected from August 1st, 2018 to March 4, 2019. Dogs included in the study had moderate to severe allergic pruritus according to the Pruritus Visual Analog Scale (PVAS) ≥ 50 mm [“Moderate Itching”; (17, 18)]. In addition, each dog had a history of allergic dermatitis at the same time in the previous year and for the 56-day duration of the study. The PVAS was designed to record the pet owner's assessment of the severity of the dog's pruritic activity during the previous 24 h. Pruritus assessments ranged from “normal dog” to “extremely severe itching”.

Investigators attributed the dog's pruritic condition to a known or presumptive diagnosis of one of the following: food allergy, contact allergy, flea allergy, atopic dermatitis, or allergic dermatitis of undetermined; all of which would be anticipated to have ongoing pruritus without intervention. Investigators were also asked to identify the dermatitis as seasonal or non-seasonal and confirm that dogs with seasonal disease had a history of allergic disease with clinical signs at the same time in previous years. Dogs were grouped by breed and size into small, medium and large/giant.

#### Inclusion Criteria

Dogs with incidental health conditions unrelated to skin disease which required treatment received the same treatment for at least 6 weeks prior to entering the study (Day 0). This treatment could not be changed for the entire period of the study (through Day 56). Similarly, dogs previously diagnosed as having cutaneous adverse food reactions and eating a hypoallergenic diet, must have started the diet at least 6 weeks prior to day 0 and remained on the same diet during the entire period of the study. For dogs receiving allergen-specific immunotherapy (ASIT), therapy was initiated at least 6 months or more prior to entering the study. If ASIT had been discontinued, that had to occur at least 8 weeks before the beginning of the study. Dogs with allergic dermatitis and secondary bacterial staphylococcal folliculitis were included; these dogs received a single injection of Convenia® (cefovecin sodium) (Zoetis, Parsippany, NJ) at label doses in addition to Cytopoint at day 0. All dogs either received an isoxazoline treatment at day 0 or were currently receiving an isoxazoline product as labeled prior to enrolling in the study. *Malassezia* (yeast) dermatitis had to be resolved prior to entry into the study. Withdrawal times for specific treatments are found in Table 1.

**Table 1:** Prohibited medications and their withdrawal time prior to study enrollment.

**Drug**	**Withdrawal time**
Janus kinase-inhibitors	1 week
Cyclosporine or tacrolimus (oral, ocular or other route)	4 weeks
Long-acting injectable corticosteroids	6 weeks
Short-acting injectable corticosteroids	4 weeks
Oral corticosteroids	4 weeks
Topical steroids/NSAID/antihistamines, including shampoos, creams, ointments, sprays, and otic and ophthalmic products	3 weeks
Antihistamines (oral or injectable)	1 week
Long-acting injectable antibiotics	3 weeks
Oral antibiotics and antifungal drugs	1 week
Miscellaneous potentially antipruritic products: Gabapentin, MAOIs	4 weeks

#### Exclusion Criteria

All breeding animals were excluded from the study as were dogs with untreated concomitant demodectic mange or untreated yeast dermatitis. Animals previously treated with Cytopoint at any time were also not eligible to be included into the study.

### Data Collection

**Figure 1:**
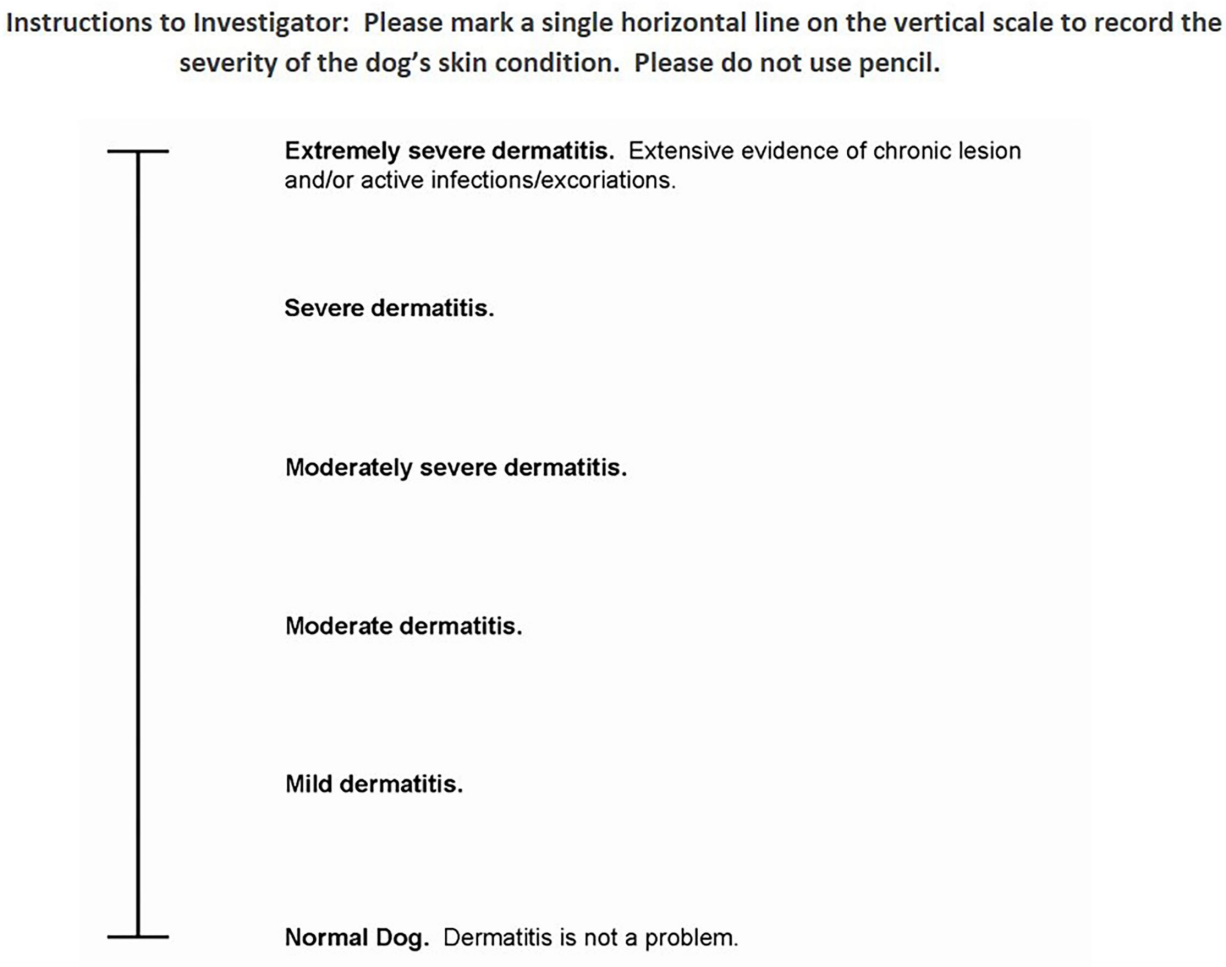
Investigator visual analog score (VetVAS).

On day 0 investigators recorded the initial body weight and administered one dose of Cytopoint (per label instructions of a minimum 2 mg/kg SQ; Zoetis, Parsippany, NJ) and an isoxazoline product to every patient. On days 0, 28 and 56 investigators performed an examination of the animals and completed a Medication and Physical examination form. Investigators confirmed if prescribed medications were still being used and documented any abnormal health condition. They also completed the Veterinarian (investigator) Visual Analog Assessment (Vet VAS; Figure 1). All dogs were examined by the same site investigator at each visit.

Prior to study data being collected by the owner, investigators ensured the familiarity of the owner with the PVAS application (created for this study; Zoetis, Parsippany, NJ). Dog owners electronically captured the severity of the pruritus using a PVAS score consistent with the paper copy version described elsewhere (17, 18). Owners were asked to complete the electronic PVAS on days 0, 1, 2, 3, 7, 14, 21, 28, 35, 42, 49 and 56. To be included in the data analysis, three criteria had to be met: all dogs had to have day 0 PVAS assessments > 50 mm, all dogs had to remain in the study until day 56, and they had to have at least 3 PVAS assessments completed over the course of the study.

### Measurements

The owner completed the first PVAS at the veterinary clinic under the supervision of the investigator. Additional scoring was completed by the owner and occurred at prescribed intervals away from the veterinary clinic.

Veterinarian Visual Analog Scale (VetVAS): This scoring system was completed on Day 0, 28 and 56 by the investigator as an assessment of the extent and severity of the dog's skin condition. Normal skin would be scored as 0 with severe to extremely severe dermatitis scored as > 85mm. This scoring system has been used in prior atopic and allergic dermatitis studies (13, 16, 19, 20) as there is no validated lesions scoring scheme for dogs with allergic dermatitis.

### Determination of Treatment Efficacy (Success Criteria)

Our objectives were to assess the efficacy of Cytopoint in reducing pruritus in dogs with allergic dermatitis of variable etiology. Treatment success for this study was defined as a ≥ 20 mm reduction in PVAS from Day 0. Secondary outcome variables included tabulation of the number of cases with reduction of PVAS below 36 mm, and a PVAS below 20 mm (“normal itch”) on days 1, 3, 7, 28 and 56.

### Statistical Analysis

All variables were summarized using SAS Proc Means (SAS 9.4, Cary, NC). No statistical inference was performed.

## Results

### Study Population

Eighty-two dogs from 8 clinics were enrolled in the study. Twenty dogs deviated from the study protocol or withdrew from the study. Two dogs achieved primary study success by Day 28 but needed a second injection of Cytopoint on Day 28 to control their ongoing pruritus (Table 2). Therefore, 62 dogs successfully completed this study; 37/62 (60% had seasonal allergies, 19/62 (30%) had nonseasonal allergies and 6/62 (10%) were not specified as seasonal or non-seasonal but had presented for allergic dermatitis within the prior year (Table 3).

**Table 2:** Reasons for patient withdrawal.

**Reason**	**Number of dogs (%)**
Inadequate data collection/owner compliance.	18 (90)
Achieved <20 mm reduction, but needed a 2^nd^ injection at day 28	2 (10)
Total	20 (100)

**Table 3:** Patient distribution.

**Age (years)**	**Number (%)**
<4	24 (39)
4–8	20 (32)
> 8	16 (26)
Not specified	2 (3)
Total	62 (100)
Presumptive diagnosis	
Atopic dermatitis	21 (34)
Pododermatitis	19 (31)
Otitis	9 (15)
Flea allergic dermatitis	6 (10)
Not specified	4 (5)
Hot spot	2 (3)
Contact dermatitis	1 (2)
Total	62 (100)
Seasonality	
Seasonal	37 (60)
Not seasonal	19 (30)
Not specified	6 (10)
Total	62 (100)

### Signalment

Twenty-nine male (46.8%) and 33 (53.2%) female dogs were enrolled. Twenty-six breeds were represented including Mixed (19 [31%]), Labrador Retriever (7 [11%]), Golden Retriever (4 [6%]), Pit Bull (4 [6%]), German Shephard (4 [6%]), Yorkshire Terrier (2 [3%]), Alaskan Malamute (2 [3%]) and 1 each of several other breeds. Fourteen (23%) dogs were classified as toy/small breeds, 19 (31%) as medium and 29 (47%) as large or giant breeds. Dogs ranged in age from 6 months to 12 yrs. old (average 5.77 yrs.) and weighed 3 to 74.5 kg. (average 24.3 kg.) (Table 3).

### Response to Treatment

The mean PVAS score on day 0 was 74.5. On day 3 the mean score was 39, by day 7 the mean score was 25.2, and by day 28 the mean score was 20.58. These PVAS scores represent a mean level of pruritus which was severe on day 0, progressing to mild on days 3 and 7 and almost normal (defined as <20) at day 28 (Table 4).

**Table 4:** Mean PVAS and treatment success.

**Variable**	**Study day**					
	**0**	**1**	**3**	**7**	**28**	**56**
PVAS, mm mean ± SD^a^ (range)	74.5 ± 12.6 (51–100)	53.1 ± 24.2 (0–90)	39.0 ± 25.7 (0–79)	25.2 ± 22.8 (0–71)	20.58 ± 21.3 (0–66)	20.2 ± 22.5 (0–76)
Treatment success, %		47	77	94	98	93
Dogs with data, *n*	62	43	47	47	50	57

*Percentage of success is calculated based on number of dogs with assessable data points*.

**Figure 2:**
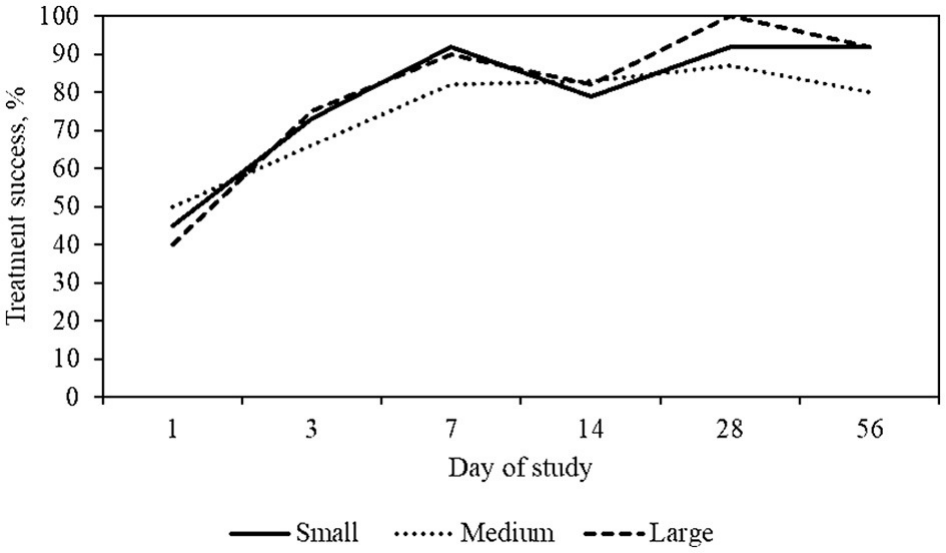
Percent treatment success, defined as >20 mm decrease in PVAS, by dog size and day of study. Small dogs include weights between 2 and 11 kg, medium dogs between 12 and 25 kg, large dogs between 26 and 55 kg.

On day 7, 47/62 dogs had assessable results and 44/47 (94%) achieved treatment success. Of these dogs with assessable results, 26/47 (55%) had a level of pruritus at or below 20 mm. By day 28 (+/– 7) a total of 50/62 dogs had assessable results and 49/50 (98%) had achieved treatment success (Table 4). Of these dogs, 32/50 (64%) dogs with assessable data had a level of pruritus at or below 20 mm, consistent with a normal dog (Table 5; Figure 2). By day 56, 62/62 (100%) of dogs achieved a PVAS 20 mm less than their day 0 PVAS on at least one timepoint during the course of the study, and 32/57 (56%) achieved a PVAS consistent with a normal dog. The mean PVAS on day 56 was 20.2 mm and at a level considered normal itch for dogs (Table 5, Figure 2).

**Table 5:** Cytopoint treatment success by three different criteria.

**Success criteria**	**Treatment success (%) on study day**					
	**0**	**1**	**3**	**7**	**28**	**56**
Decrease by 20 mm in PVAS from day 0^a^	0	47	77	94	98	93
<36 mm (COSCAD)^b^	0	21	40	68	70	63
<20 mm (normal itch)^c^	0	12	32	55	64	56
Dogs with data, n	62	43	47	47	50	57

^a^*Cosgrove et al. (19)*.

^b^*Olivry T., et al. (21)*.

^c^*PVAS normal itch*.

Over the course of the study, for each body weight grouping, there was an increase over time in the number of dogs achieving treatment success. A description of treatment results is detailed in Table 6.

**Table 6:** Cumulative Cytopoint treatment success for small, medium and large dogs.

**Category**	**Weight (kg)**	**N**	**Cumulative treatment success (%) on study day**		
			**3**	**7**	**56**
Small	2 to 11	14	64	93	100
Medium	12 to 25	19	68	89	100
Large	26 to 55	29	77	88	100
All	2 to 55	55 to 62^a^	71	89	100

^a^*By day 56, the number of dogs with data ranged from 55 to 62*.

Ten dogs (10/62 or 16%) received an antibiotic treatment Convenia® (cefovecin sodium) on day 0. By day 14, 8/10 (80%) of this group had achieved treatment success for PVAS. By day 28, when the dogs were reexamined, 10/10 (100%) of these dogs had achieved treatment success for PVAS and their pyoderma had resolved.

Ten (10/62) dogs entered the study with the most severe pruritus (PVAS > 90). By day 3, 10/10 (100%) of these dogs had achieved treatment success. In addition, 6/10 (60%) achieved a PVAS <20 mm on days 28 and 56.

This study included 6 dogs with flea allergic dermatitis. Half (3/6) of the dogs had live fleas noted at their initial examination. All dogs received an isoxazoline treatment and when reexamined on day 28, flea infestations were not detected. The mean PVAS score on day 0 was 79.7 and by day 7, 5/6 dogs had available data with a mean PVAS score of 20.6 and had achieved treatment success. By day 14 all 6 dogs had assessable data with a mean PVAS score of 15.8. On day 14, 100% of the dogs achieved treatment success. At day 28, there was a mean PVAS of 17. In addition, by day 14, 5/6 (83%) of the dogs achieved a PVAS <20. At day 28, there was a mean PVAS of 17, 6/6 or 100% of these dogs had achieved treatment success and 5/6 (83%) had achieved a PVAS <36 mm.

The mean VetVAS score on day 0 was 28.0 mm (SD ± 9.6) consistent with moderate dermatitis. On day 28, 54/59 (92%) dogs achieved a 50% reduction in their VetVAS score with a mean VetVAS of 6.0 mm (SD ± 7.1) and by day 56, 45/58 (78%) maintained a 50% reduction in their VetVAS scoring with a mean score of 6.6 mm (SD ± 8.9). This represents an improvement from moderate to mild dermatitis, with scores approaching a level consistent with a normal dog (Figure 1).

## Discussion

The objective of this study was to demonstrate the efficacy of Cytopoint for the treatment allergic dermatitis and the pruritus which is often associated with allergic dermatitis. The cohort of dogs had a variety of allergic dermatoses, some with only one suspected allergy and others with a combination. In a recent study, it was suggested a more rigorous diagnostic criteria assessed by dermatologists would likely alter the diagnoses distribution (16), but this study was intended to reflect primary practice methods and attitudes, thus reflecting how general practice veterinarians would diagnose and treat dogs with pruritus associated with allergic dermatitis of various etiologies.

In this study, 47% of the dogs achieved treatment success within 24 h of treatment and 77% had achieved treatment success by day 3. These results are consistent with the pharmacokinetic profile previously reported for Cytopoint, where the onset of efficacy is described within 1 day of administration [39% for 2.0 mg/kg treatment group; (13)]. In a retrospective study, (15) noted clinical improvement in 56% of dogs with allergic dermatitis within the first 24 h.

Allergic pruritus affects dogs of all ages and sizes. In this study, Cytopoint effectiveness was not related with the type of allergic dermatitis, age of dog. However, in a previous retrospective study, 87% of the animals were reported to be lokivetmab treatment successes, with greater reduction of pruritus reported in large dogs and dogs with greater pruritus intensity (15). In the current study, similar allergic dermatitis and the associated pruritus was controlled among all treated dogs, independent of dog size and pruritus severity. To the authors' knowledge, the response difference by weight has not been noted in other studies. However, differences observed in success rates between Souza et al. (15) and our study could be due to cofactors such as owner compliance, concomitant diseases, possible differences in severity of disease seen in referral populations, treatment protocols, etc. that are carried out differently in retrospective and prospective studies.

Another method to assess efficacy, (18) suggested a pruritic threshold for a normal dog to be <20 on the PVAS scale. Using this more stringent criteria, 55% of animals in this study reached this threshold by day 7 and 64% by day 28. This is in line with the results from a lokivetmab prospective study (16) which found 45.5% of dogs achieved this criterion by day 28.

The VetVAS used in this study has been employed in prior oclacitinib and Cytopoint research (13, 19). While this scale has not been formally validated, it has provided insight to the degree of skin lesions and dermatitis observed by investigators. For the dogs in this study, all but 3 at day 28 and all but 1 at day 56 had at least a 50% reduction in their VetVAS supporting a reduction in skin inflammation and dermatitis, consistent with prior lokivetmab studies (16).

Uncontrolled pruritic activity may lead to the breakdown of skin barriers and secondary infection. In this study, a proportion of the dogs presented with concomitant pyoderma. For these dogs, treatment with Convenia(cefovecin sodium) and Cytopoint provided treatment success with resolution of pyoderma and reduction of pruritus, providing support for the use of Cytopoint for dogs presenting with allergic pruritus with secondary pyoderma.

In a similar way, dogs with flea allergy dermatitis showed positive outcomes when treated with Cytopoint and an isoxazoline anti-parasiticide. In this study, the decision to follow CAPC guidelines which recommend all dogs receive monthly flea treatment was undertaken, thus all dogs received an isoxazoline treatment (22). When dogs develop flea allergy dermatitis, control of the flea burden is critical, but depending on circumstances, reaching 100% flea control may take weeks to months. Control of the ongoing pruritus is key to preventing additional sequela like secondary skin lesions. All flea allergic dogs in this study had a notable and rapid reduction in their pruritus, with 83% having pruritus levels return to normal dog levels by day 14. These results support the use of Cytopoint to manage pruritus in flea allergic dogs while steps are taken to control (or eliminate) fleas.

A total of 20 dogs were withdrawn during the study, with 18 withdrawn due to poor owner compliance and deviation from the protocol. In our study some owners abandoned the treatment and did not return to the clinic. The reasons for owners not returning per study protocol is unknown – amongst those may have been dogs where treatment was, or was not, effective.

This study design presents limitations consistent with an open, uncontrolled study including unblinded study investigators and the lack of comparison with a control placebo group or other antipruritic therapies. Despite these limitations, these results are consistent with a recently completed lokivetmab randomized controlled clinical trial (16) and are intended to provide clinicians with a body of evidence supporting additional treatment options when considering a patient with allergic dermatitis.

In conclusion, allergic dermatitis including atopic dermatitis is often a lifelong disease and typically requires ongoing therapy for long-term management. This study supports the use of Cytopoint as a treatment option in dogs presenting not only with atopic dermatitis but also with other allergic dermatoses.

## Data Availability Statement

The raw data supporting the conclusions of this article will be made available by the authors, without undue reservation.

## Ethics Statement

The animal study was reviewed and approved by Zoetis ethics review board. Written informed consent was obtained from the owners for the participation of their animals in this study.

## Author Contributions

MG collected, analyzed, interpreted the allergic disease data, and was the primary contributor to the writing of the manuscript. AH analyzed, interpreted the allergic disease data, and reviewed the manuscript. MV-H was a major contributor to the writing of the manuscript. DA analyzed the data. MM was a contributor to the writing of this manuscript. All authors contributed to the article and approved the submitted version.

## Funding

This study was funded by Zoetis.

## Conflict of Interest

MG, AH, MV-H, DA, and MM were employed by Zoetis LLC. This study received funding from Zoetis LLC. The funder had the following involvement with the study: study design, collection, analysis, interpretation of data, the writing of this article and the decision to submit it for publication.

## Publisher's Note

All claims expressed in this article are solely those of the authors and do not necessarily represent those of their affiliated organizations, or those of the publisher, the editors and the reviewers. Any product that may be evaluated in this article, or claim that may be made by its manufacturer, is not guaranteed or endorsed by the publisher.
